# Infectious Bursal Disease Virus-Host Interactions: Multifunctional Viral Proteins that Perform Multiple and Differing Jobs

**DOI:** 10.3390/ijms18010161

**Published:** 2017-01-14

**Authors:** Yao Qin, Shijun J. Zheng

**Affiliations:** 1 State Key Laboratory of Agrobiotechnology, Beijing 100193, China; qyao_cau@163.com; 2 Key Laboratory of Animal Epidemiology and Zoonosis, Ministry of Agriculture, Beijing 100193, China; 3 College of Veterinary Medicine, China Agricultural University, Beijing 100193, China

**Keywords:** infectious bursal disease virus (IBDV), immunosuppression, cellular target

## Abstract

Infectious bursal disease (IBD) is an acute, highly contagious and immunosuppressive poultry disease caused by IBD virus (IBDV). The consequent immunosuppression increases susceptibility to other infectious diseases and the risk of subsequent vaccination failure as well. Since the genome of IBDV is relatively small, it has a limited number of proteins inhibiting the cellular antiviral responses and acting as destroyers to the host defense system. Thus, these virulence factors must be multifunctional in order to complete the viral replication cycle in a host cell. Insights into the roles of these viral proteins along with their multiple cellular targets in different pathways will give rise to a rational design for safer and effective vaccines. Here we summarize the recent findings that focus on the virus–cell interactions during IBDV infection at the protein level.

## 1. Introduction

The functional integrity of the immune system is essential to prevent invasions of pathogens and maintain a state of health. It is difficult for the host to control the contagious diseases when the pathogens directly breakdown the immune system. Infectious Bursal Disease Virus (IBDV) is one of these formidable opponents to the host defense system, attacking and destroying the developing B-lymphocytes in the bursa of Fabricius (BF) [1], the central immune organ for the development and maturation of B cells and the generation of diverse antibody repertoire in young chickens [2]. Chickens of 3 to 6 weeks, at the maximal stage of BF development, are susceptible to IBDV infection [1,3]. The mortality is determined by multiple factors including the virulence of IBDV, the dose of infection, the age and breed of chickens, and the passive immunity as well [4]. In the case of very virulent IBDV (vvIBDV) infection, 50% to 100% mortality in young chickens can be observed [5,6,7]. Like most RNA viruses that develop the “bite and run” strategy [8], the acute IBDV infection causing clinical manifestations lasts for only 3 to 4 days. Although rapid recovery from Infectious Bursal Disease (IBD) is common in survival chicks, the damage to BF is irreversible and leads to immunosuppression, followed with increased susceptibility to other diseases and the failure of vaccinations [9]. Thus, IBDV infection is a serious problem threatening poultry industries across the globe.

The interactions between IBDV and host leading to the pivotal steps of the virus life cycle including virus entry, uncoating, replication, assembly and release, are important implications for developing the strategies to control IBD. On the other hand, the massive depletion of B cells caused by IBDV-induced apoptosis is the major reason to cause immune dysfunction, which is also partially due to the decreased phagocytotic activity of monocytes/macrophages [1,10] and the diminished response to mitogen activation of T cells as well [11,12]. IBDV needs to accomplish the replication and maturation before it breaks down the host cell. The answers regarding how the virus controls the apoptotic responses and how the immune system is impaired by IBDV can be found in the interactions of IBDV with their cellular targets. The total number of viral proteins encoded by the IBDV genome is relatively limited. Therefore, it is very likely that these proteins have multiple functions and are able to bind to different cellular factors during IBDV infection. Based on the applications of protein–protein interaction detection methods such as yeast two-hybrid and pull-down assays, the IBDV–host interactions in different molecular events regarding endocytosis, apoptosis, autophagy and cytokine productions are found at protein levels. However, further efforts are required to complete the whole picture about the network of the IBDV–host interaction and biological relevance. The breakthrough findings of the IBDV–host binding sites associated with IBDV-triggered impairment of the host defense contribute to the key solutions to the immunosuppression induced by live vaccines. This review is mainly focused on the recent findings about the interactions of the viral proteins with their cellular targets in the important molecular events during IBDV infection. 

## 2. Virus Characteristics

### 2.1. Virus Structure of IBDV

IBDV is an *Avibirnavirus* belonging to the Birnaviridae family, which is composed of non-enveloped and icosahedral viruses enclosing two segments of double-stranded RNA (A and B) [13]. Whereas the short RNA, segment B (2.8 kb) encodes VP1 (97 kDa), an RNA-dependent RNA polymerase (RdRp) [14,15], segment A, the larger one (3.17 kb) contains two partially overlapping open reading frames (ORFs) that encode the major components of the virus [16,17]. The first ORF encodes the nonstructural viral protein VP5 (17 kDa) and the second one encodes a polyprotein precursor (pVP2–VP4–VP3, 110 kDa) that can be cleaved by VP4 (28 kDa) in *trans* to release pVP2 (512 residues, 54.4 kDa) and VP3 (32 kDa) [18]. Both VP4 and the puromycin-sensitive aminopeptidase (PurSA) cleave the pVP2 at its C-terminus to generate the intermediate pVP2 (452 residues) [19], which is further processed by VP2 itself to generate the mature VP2 (441 residues) [20,21]. VP3 acts as a scaffold protein that binds both the viral double-stranded RNA and VP1 [22]. VP2 and VP3 are the major structural proteins, constituting 51% and 40% of the virion, respectively [23]. The mature VP2 with a variable amount of pVP2 (452 residues) and VP3 assemble the single shelled capsid of IBDV [21]. The released peptides arising from the cleaved pVP2 are also assembled in the virus, contributing to the virus viability and cell membrane perforation [24]. 

### 2.2. Genetic Variation of IBDV

A high genetic mutation rate is the key feature of RNA viruses [8]. The hyper-variable region (HVR) of IBDV is located in the *vp2* gene (206 aa to 350 aa), which is responsible for the antigenic variation since VP2 induces neutralizing antibodies [25]. IBDV takes advantage of the genetic flexibility to achieve antigenic variations, which help itself to escape immune clearance and quickly adapts to the change of environment. The emergence of very virulent IBDV (vvIBDV) strains was reported in the late 1980s [5]. The putative amino acids responsible for high virulence were at Gln253, Asp279 and Ala284 in VP2 [26]. The residues 249 and 256 of VP2 were also found involved in the replication efficiency and virulence of IBDV [27]. In addition to the mutations in HVR of VP2, genetic reassortment events and homologous recombination within segments also contribute to the variation of IBDV [28,29,30,31]. A molecular epidemiology study of IBDV isolates from seven provinces in southern China during 2000–2012 showed that the majority of the isolates (85.71%, 78/91) were identified to be naturally reassorted strains [32]. The recombination leading to the variation of IBDV virulence can be evidenced in the different isolates having segment A region consistent with vvIBDV [33,34]. With the intensive uses of live vaccines, the number of vvIBDV strains and their reassortants have continuously increased and the strains have become epidemic and posed a great threat to the poultry industry with the current anti-IBDV vaccination strategy [35,36], making the prevention and control of IBD more challenging. 

## 3. Host and Target Cells

There are two serotypes of IBDV, serotype I and serotype II [37]. However, chickens only develop IBD after the infection by serotype I IBDV strains [4]. Some other birds including pigeon and guinea fowl can be infected by IBDV without apparent pathology [38]. Chickens, especially young chicks at the age of 3 to 6 weeks, are the selected hosts for the serotype I virus [3]. In the case of vvIBDV infection, the age susceptibility is extended which covers the entire growing period in broilers [4]. In addition, it was reported that chickens infected with IBDV at the age of 14 days suffered from greater bursal atrophy and had higher viral RNA copy numbers than those infected on the day of hatching [39]. Furthermore, different genetic backgrounds of chicken breeds may have different impacts on the early immune responses to IBDV infection [40,41]. It was found that light breeds showed higher mortality than the heavier breeds [42,43]. Moreover, the layer-type chickens were more susceptible to vvIBDV than broiler-type chickens, both in conventional status and specific-pathogen-free (SPF) status [41,44]. Serotype II IBDV strains isolated from turkeys and Peking ducks are avirulent to chickens [37].

The specific tropism of IBDV to developing B cells in the BF has been well-documented, and most of the target B cells are immunoglobulin M positive (IgM^+^) cells [1]. IBDV also invades and replicates in the cells of monocyte–macrophage lineage in a persistent manner [45], which impedes the phagocytic activity of macrophages and facilitates virus dissemination [10]. In addition, the virus can infect chicken bone marrow-derived dendritic cells [46]. 

## 4. Cellular Receptors and the Key Elements Associated with the Entry of IBDV

Like other non-enveloped viruses lacking outer membrane, IBDV is unable to utilize direct membrane fusion to enter the target cells [47], and the mechanism that facilitates the invasion of IBDV is still unclear. The first step of IBDV infection is the attachment of virus to the cellular membrane surface of target cells by binding to the specific receptors. Several proteins on the cell surface have been shown to be involved in the entry of IBDV into the cell, which are mostly discovered in the host cellular proteins that bind to the viral particles or VP2, the major component of the viral capsid. 

Surface immunoglobulin M (sIgM) is the first reported cellular receptor for IBDV [48], and the further study showed that λ light chain of sIgM can bind to the virus particles in a virulence-independent manner [49]. These data support the previous report that implies the IgM^+^ B-cells serve as the major targets of IBDV [1]. Chicken heat shock protein 90 (HSP90) in the surface of DF-1 cell membrane was found to interact with IBDV particle or VP2-subviral particle (SVP), acting as a putative receptor [50]. Besides, the Ile-Asp-Ala (IDA) sequence within the VP2 P domain was identified as the functional ligand motif to α4β1 integrin based on a multiple alignment [51]. The α4β1 heterodimer is highly abundant in immature lymphocytes [52], which is in accordance with the age tropism of IBDV. The downstream of IBDV-α4β1 binding is related to the c-Src phosphorylation that activates the Akt-RhoA–actin rearrangement cascade for endocytotic internalization of IBDV [53]. The crucial role of actin rearrangement in IBDV internalization can also be evidenced by the finding that the entry of IBDV involved macropinocytosis and trafficking to early endosomes in a Rab5-dependent manner [54]. However, there is still a lot of work to elucidate the exact role of VP2-α4β1 interaction in IBDV entry.

The entry strategies employed by non-enveloped virus have not been fully understood so far [47]. Nevertheless, the cellular membrane perforation and conformational alteration were well-documented to be involved in the essential steps for non-enveloped virus to cross the membrane barrier [47]. In the case of IBDV infection, Pep46, a capsid-associated peptide generated from the C-terminus of pVP2 by VP4 cleavage, showed membrane permeabilization activity that deformed the endosomal membrane by forming pores [55]. The release of Pep46 from viral capsid to cellular endosome was dependent on the low calcium concentration environment [55]. These findings suggest that the entry of IBDV, via the pores in the endosomal membrane formed by Pep46, needs endocytosis to release Pep46 first. Interestingly, this hypothesis is partially proved by the successive study, which showed that IBDV was endocytosed and transported to the V-ATPase positive vesicles for uncoating [56]. Furthermore, a recent study indicated that Annexin II (Anx2), a calcium- and phospholipid-binding protein that has shown to function in membrane traffic within endocytosis, exocytosis and cell adhesion [57,58,59], also acts as cell surface receptor that binds to IBDV VP2 [60]. Taken together, these findings suggest that endocytosis is required for IBDV entry and internalization, followed by the release of Pep46 from the viral capsid to the endosome, which induces endosome permeabilization facilitating the escape of virus into the cytosol.

## 5. Suppression of Host Immune Responses

The immunosuppression caused by IBDV infection is complex. On one hand, the massive depletion of B cells during IBDV infection directly breaks down the generation of diverse antibody repertoire so that the acquired immune system fails to response to other pathogens as mentioned before. On the other hand, the components of IBDV can inhibit the host innate immune response besides B cell apoptosis.

### 5.1. Apoptosis Leads to the Depletion of Lymphoid Cells

Apoptosis contributes to the depletion of lymphocytes [61,62,63]. In addition to the rapid loss of B cells in the BF, a high level of apoptosis is found in chicken peripheral blood lymphocytes during IBDV infection [61]. However, apoptosis was regarded as a critical cellular defense mechanism against viral invasion since the apoptosis of infected cells limits viral replication and spread [64,65]. In contrast, it seems that apoptosis occurring in IBDV infection is initiated or manipulated by the virus rather than the consequence of antiviral responses of host, because the host cell apoptosis contributes to the viral release late in the life cycle [66], which is obviously beneficial to IBDV rather than to the host. Thus, the timing of the induction of apoptosis needs to be controlled. Both the structural protein VP2 and the nonstructural protein VP5 are the weapons employed by IBDV to induce the programmed cell death process [66,67].

**Figure 1:**
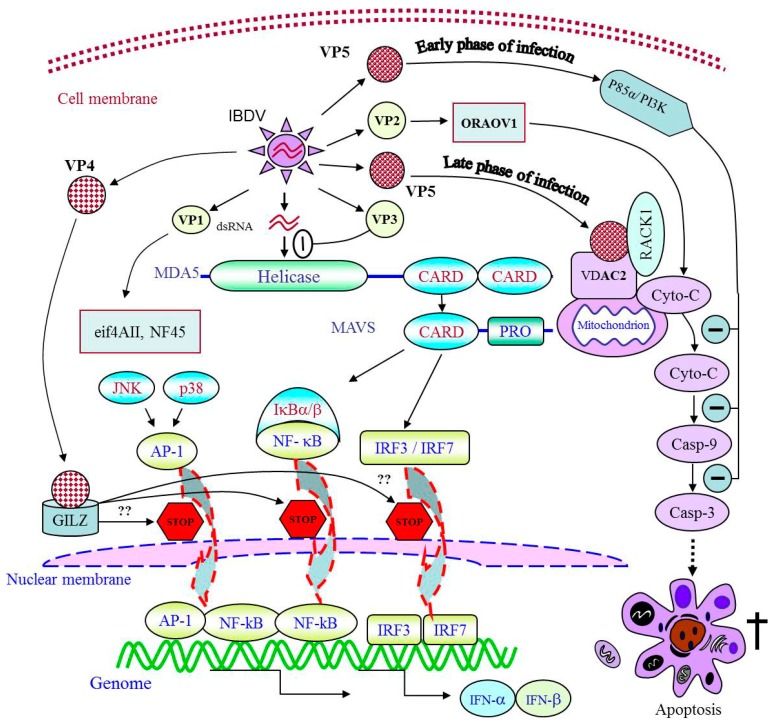
Schematic illustration of apoptosis and immunosuppression induced by viral proteins via interaction with cellular targets; Once expressed inside host cells, the viral proteins of IBDV, via interaction with cellular targets, play different roles in facilitation of IBDV replication; NF-kB: nuclear factor kappa enhancer binding protein; JNK: c-Jun-N-terminal kinase; AP-1: activating protein-1; IRF: interferon regulatory factor; PI3K: phosphoinositol-3 kinase; MAVS: mitochondrial antiviral signaling protein; CARD: caspase activation and recruitment domain; eif: eukaryotic initiation factor; ORAOV1: oral cancer overexpressed 1; GILZ: glucocorticoid-induced leucine zipper; VDAC2: voltage-dependent anion channel 2; RACK1: receptor of activated protein kinase C1; IFN: interferon; Cyto-C: cytochrome c; Casp: caspase; dsRNA: double-stranded RNA.

VP2 was the first identified apoptotic inducer in the IBDV infection, which exhibits cytotoxicity in host cells and a variety of mammalian cell lines as well [67]. It suggests that the mechanism underlying VP2-induced apoptosis includes conservative signals to activate the cell death. A strong shut-off effect of VP2 on cellular protein synthesis was proved to be located upstream of Bcl-2 and accompanied with the activation of protein kinase R (PKR) pathway [67,68]. Moreover, an increased reactive oxygen species (ROS) level was observed in the cells infected with IBDV [69]. Several reports have related the oxidative stress to PKR pathway activation [70,71]. Therefore, the accumulated ROS may also take part in the cell death through the activation of the PKR-pathway. The activations of caspase 3 and caspase 9 were also reported in IBDV-induced apoptotic cells [72]. Our unpublished data show that VP2 triggers apoptosis via interacting with oral cancer overexpressed 1 (ORAOV1) (Figure 1) [73]. To investigate the molecular mechanism of VP2-induced apoptosis, we employed the yeast two-hybrid screening and immunoprecipitation assays and found that ORAOV1 interacts with IBDV VP2. ORAOV1 was identified as a pivotal regulator of cancer cell growth [74,75], and ROS production [76,77]. Knockdown of ORAOV1 expression can trigger apoptotic pathways including the release of cytochrome c and the cleavages of caspase 3, caspase 8 and caspase 9 [75]. However, the exact molecular mechanism underlying VP2-induced apoptosis needs further clarification.

The role of VP5 as an apoptotic inducer can be evidenced by the decreased level of cell death in the cells infected with an VP5-deficient IBDV strain [78,79], as well as severe cellular DNA fragmentation induced by transient expression of VP5 [66]. Furthermore, our previous findings confirmed the dominant role of VP5 in IBDV-induced apoptosis mediated by the interaction of VP5 with voltage-dependent anion channel 2 (VDAC2) [80] and receptor of activated protein kinase C1 (RACK1) [81]. We found that VDAC2 was indispensable to the release of cytochrome c and the activation of caspase 9 or 3, which led to apoptosis during IBDV infection, while RACK1, an antiviral protein, was suggested to be on behalf of the counteractions of host (Figure 1). Liu and Vakharia proposed that VP5 might play a role as anti-apoptotic protein at an early stage of infection [72]. In addition, it was reported that VP5 might act as an anti-apoptotic molecule by binding to p85α subunit of phosphoinositol-3 kinase (PI3K) early during IBDV infection (Figure 1) [82]. These results suggest that VP5 as an anti-apoptotic protein is an important factor to support viral replication at the early stage of IBDV infection. Our data show that VP5 induces apoptosis by binding to VDAC2 at the late stage of IBDV infection to facilitate viral release [80]. Furthermore, Qin and his colleague reported that VP5-deficient mutant IBDV caused reduced bursal lesion of SPF chickens compared to the parental virus, indicating that VP5 induces tissue damage in vivo [83]. The paradoxical findings of the role of VP5 in cell apoptosis may be due to the different time points of observation. The anti-apoptotic activity of VP5 was observed at 8 or 12 h post-infection [82] while VP5 induced apoptosis were found after 24 h post-infection [80]. Thus, we propose that IBDV VP5 interacts with different cellular targets, and its multi-functions at different stages of IBDV infection depend on the binding affinity of VP5 with its targets and the quantity of VP5 in the cytoplasm as well. Mathematic biology may be required to reveal multiple roles of VP5 during IBDV infection.

An anti-apoptotic activity of VP3 was also reported [68]. The expression of VP3 impeded the phosphorylation of both PKR and eukaryotic initiation factor 2 (eIF2) resulting in the inhibition of PKR-mediated apoptosis. It indicates that VP3 may be employed by IBDV to moderate VP2-induced apoptosis, preventing the activation of apoptosis before the virus can accomplish the replication and be ready to release.

### 5.2. Key Elements That Impair the Innate Immune Response

Apoptosis in B cells is not the only cause for the suppression of host immune responses because IBDV also retards the antigen presentation pathway. It has been shown that IBDV infection leads to the robust expression of proinflammatory cytokine transcripts along with a decrease in macrophage numbers, suggesting that IBDV may lead to a reduction of resident macrophages in vivo [84]. It has been reported that IBDV infection impairs chicken bone marrow-derived dendritic cells, with low expression level of co-stimulatory molecules including CD40 and CD86 [46]. Furthermore, IBDV infection can diminish the response to mitogen activation of T cells [1], which was proposed to be related to the production of chicken interferon-gamma (IFN-γ) [11]. The impairment of cytokine productions induced by IBDV was also evidenced by the fact that IBDV infection interfered with the transcription of chicken type I and II interferon mRNAs [85]. However, it remains unclear how IBDV triggers these changes, besides the loss of B cells, of the immune responses against the infection, aggravating the immunosuppression of the chicken. So far there are two virus components (VP4 and VP3) that have been proven to be directly involved in the suppression of the innate immune response against IBDV.

VP4, known as the viral protease to cleave the polyprotein, acts as an essential viral component to suppress type I interferon expressions via binding to glucocorticoid-induced leucine zipper protein (GILZ) [86], a cellular factor that inhibits the activation of nuclear factor kappa enhancer binding protein (NF-κB) [87]. As type I interferon expression is regulated by transcriptional regulator NF-κB [88], VP4-induced suppression of the type I interferon response might result from the inhibitory effect of VP4 on the activation of NF-κB (Figure 1). The translocation of NF-κB to the nucleus, activation of interferon regulatory factors 3/7 (IRF3/7) and/or activator protein-1 (AP-1) cooperate to stimulate transcription of IFN-α/β [89,90]. Furthermore, GILZ can inhibit the AP-1 activation [91]. Thus, it is reasonable to suggest that the downstream cascade of the interaction of VP4 and GILZ might be related to the activation of AP-1 or/and IRF3/7 (Figure 1). Moreover, the double-stranded RNA (dsRNA)-binding ability of VP3 that stabilizes the virus structure, may also contribute to the blockage of viral dsRNA interacting to MDA5 (Figure 1), a well-known pattern recognition receptor that detects viral RNA in the cytoplasm and initiates the innate immune response via interaction with MAVS, a mitochondrial antiviral signaling protein [92]. The signaling domains of MDA5, named CARD, must form homo-oligomers in order to bind to MAVS, and their bindings induce filament formation of MAVS, for downstream signal transduction [93]. These findings suggest that IBDV survives in host cells from innate immune response by at least two strategies: one is to suppress the type I interferon expressions via VP4 binding to GILZ, and the other is to escape from the innate immunity via VP3 blocking the recognition of viral dsRNA by MDA5.

Autophagy, controlled by mechanistic target of rapamycin (mTOR) kinase-dependent signaling pathway [94], is closely connected to the innate immunity [95]. Recently, it was demonstrated that the interaction of VP2 with virus receptor Heat shock protein 90 (HSP90AA1) was able to trigger the autophagy directly via AKT-mTOR pathway [96]. VP2 interfered with the binding between the cytoplasmic HSP90AA1 and AKT which is important to maintain the AKT kinase activity [97]. The disassociation of phosphorylated AKT from HSP90AA1 was found to be responsible for dephosphorylation of mTOR which then activated autophagosome formation. The autophagy triggered by IBDV was proposed to be the host defensive response to the infection since it inhibited the virus replication [96]. However, it still remains largely unknown how the cellular factors of the innate immune system interact with the virus to fight against IBDV infection.

## 6. Cellular Factors That Affect the Replication of IBDV

The identification of cellular factors involved in the viral replication contributes to the development of strategies to interfere with viral infection. RNA viruses need to hijack the translation apparatus for amplification of the viral genome. Therefore, examination of the host translation initiation factors recruited by the virus is a good start to identify the cellular proteins that have impacts on viral replication. VP1 is the RNA-dependent RNA polymerase of IBDV, serving as the major bait protein to screen the cellular targets that regulates the viral replication. The carboxy-terminal domain of translational eukaryotic initiation factor 4AII is the first reported host translation initiation factor that binds to VP1 (Figure 1) [98]. Furthermore, nuclear factor NF45, an RNA binding protein that regulates gene expression, was identified to be suppressor of IBDV replication interacting with VP1, VP2 and VP3 [99].

In addition to translation apparatus, the cellular factors recruited by viruses in other pathways including antiviral response, protein modification and transportation, are also important for the viral replication process. Chondroitin sulfate *N*-acetylgalactosaminyltransferase-2 (CSGalNAcT2), a type II transmembrane protein in Golgi apparatus, was reported to be associated with IBDV VP2 and beneficial for IBDV replication [100]. CSGalNAcT2 may locate VP2 in Golgi apparatus for virus glycosylation or assembly. A recent study showed that Cyclophilin A (CpA) interacted with IBDV VP4 and the overexpression of CpA inhibited the replication of IBDV [101]. It proposed that CpA may influence the enzyme function of VP4 via the peptidyl-prolyl *cis–trans* isomerase (PPIase) activity of CpA and triggering both the T cell activation and the IFN-I or interleukin-2 (IL-2) response. In contrast, the interaction of GILZ with VP4 enhanced the IBDV replication since GILZ suppressed the immune response via inhibiting type I IFN expression [86]. 

## 7. Conclusions

In order to complete the life cycle in the host, viruses have refined different strategies to overcome the host defense and hijack cellular factors in different pathways. Unlike the DNA viruses with large genome, RNA viruses with limited length of genome are unable to have a large arsenal of virulence factors. Therefore, proteins of RNA viruses are multifunctional and bearing multiple tasks. The virulence factors of IBDV perform different jobs in host cells to facilitate viral replication, such as the capsid protein VP2 as an apoptotic inducer, and the virus protease VP4 as a suppressor to the innate immunity. The studies of IBDV–host interactions reveal how these viral proteins perform their differing tasks. VP5 inhibits apoptosis of host cells via interaction with p85α subunit of PI3K early after IBDV infection to allow sufficient time for IBDV replication. As apoptosis is required for the spread of IBDV, VP5 induces apoptosis via interaction with VDAC2 by intrinsic apoptotic pathway at a late phase of IBDV infection. Meanwhile, apoptosis induced by VP2 via interaction with ORAOV1 may also help IBDV spread according to our unpublished data. RACK1 favors IBDV growth by inhibiting apoptosis via interaction with both VDAC2 and VP5. To accomplish the replication, IBDV interferes with the antiviral innate immune activation and hijack the host transcription factors. VP4 suppresses expression of type I interferon in host cells via interaction with GILZ, which stops transcriptional regulators NF-κB from initiating expression of type I interferon in favor of IBDV replication. VP3 inhibits MDA5 recognition of dsRNA of IBDV to help IBDV evade the immune responses of host against IBDV infection. More efforts will be required to elucidate the exact roles of IBDV components in the pathogenesis of IBDV infection.

The destruction of the immune system by IBDV dampens immune responses to pathogenic infections. The recent breakthrough findings of interactions between IBDV and its cellular targets unveil the multiple functional motifs of IBDV proteins responsible for immunosuppression and apoptosis, providing insights into the development of safer and more effective vaccines.

## References

[1] Sharma J.M., Kim I., Rautenschlein S., Yeh H. (2000). Infectious bursal disease virus of chickens: Pathogenesis and immunosuppression. Dev. Comp. Immunol..

[2] Nera K., Kylaniemi M.K., Lassila O. (2015). Bursa of Fabricius.

[3] Mahgoub H.A. (2012). An overview of infectious bursal disease. Arch. Virol..

[4] Ingrao F., Rauw F., Lambrecht B., van den Berg T. (2013). Infectious Bursal Disease: A complex host-pathogen interaction. Dev. Comp. Immunol..

[5] Chettle N., Stuart J.C., Wyeth P.J. (1989). Outbreak of virulent infectious bursal disease in East Anglia. Vet. Rec..

[6] Stoute S.T., Jackwood D.J., Sommer-Wagner S.E., Crossley B.M., Woolcock P.R., Charlton B.R. (2013). Pathogenicity associated with coinfection with very virulent infectious bursal disease and Infectious bursal disease virus strains endemic in the United States. J. Vet. Diagn. Investig..

[7] Annamalai A., Ramakrishnan S., Sachan S., Kumar B.S., Sharma B.K., Kumar V., Palanivelu M., Varghese B.P., Kumar A., Saravanan B.C. (2016). Prophylactic potential of resiquimod against very virulent infectious bursal disease virus (vvIBDV) challenge in the chicken. Vet. Microbiol..

[8] Vidalain P.O., Tangy F. (2010). Virus-host protein interactions in RNA viruses. Microb. Infect..

[9] Withers D.R., Young J.R., Davison T.F. (2005). Infectious bursal disease virus-induced immunosuppression in the chick is associated with the presence of undifferentiated follicles in the recovering bursa. Viral Immunol..

[10] Lam K.M. (1998). Alteration of chicken heterophil and macrophage functions by the infectious bursal disease virus. Microb. Pathog..

[11] Rauw F., Lambrecht B., van den Berg T. (2007). Pivotal role of ChIFNγ in the pathogenesis and immunosuppression of infectious bursal disease. Avian Pathol..

[12] McNeilly F., Walker I., Allan G.M., Adair B.M. (1999). Bursal lymphocyte proliferation in the presence of phorbol myristate acetate: Effect of IBDV strains on the proliferation response. Avian Pathol..

[13] Azad A.A., Barrett S.A., Fahey K.J. (1985). The characterization and molecular cloning of the double-stranded RNA genome of an Australian strain of infectious bursal disease virus. Virology.

[14] Macreadie I.G., Azad A.A. (1993). Expression and RNA dependent RNA polymerase activity of birnavirus VP1 protein in bacteria and yeast. Biochem. Mol. Biol. Int..

[15] Von Einem U.I., Gorbalenya A.E., Schirrmeier H., Behrens S.E., Letzel T., Mundt E. (2004). VP1 of infectious bursal disease virus is an RNA-dependent RNA polymerase. J. Gen. Virol..

[16] Spies U., Muller H., Becht H. (1989). Nucleotide sequence of infectious bursal disease virus genome segment A delineates two major open reading frames. Nucleic Acids Res..

[17] Kibenge F.S., McKenna P.K., Dybing J.K. (1991). Genome cloning and analysis of the large RNA segment (segment A) of a naturally avirulent serotype 2 infectious bursal disease virus. Virology.

[18] Lejal N., Da C.B., Huet J.C., Delmas B. (2000). Role of Ser-652 and Lys-692 in the protease activity of infectious bursal disease virus VP4 and identification of its substrate cleavage sites. J. Gen. Virol..

[19] Irigoyen N., Caston J.R., Rodriguez J.F. (2012). Host Proteolytic Activity Is Necessary for Infectious Bursal Disease Virus Capsid Protein Assembly. J. Biol. Chem..

[20] Irigoyen N., Garriga D., Navarro A., Verdaguer N., Rodriguez J.F., Caston J.R. (2009). Autoproteolytic activity derived from the infectious bursal disease virus capsid protein. J. Biol. Chem..

[21] Saugar I., Luque D., Oña A., Rodriguez J.F., Carrascosa J.L., Trus B.L., Castón J.R. (2005). Structural Polymorphism of the Major Capsid Protein of a Double-Stranded RNA Virus: An Amphipathic α Helix as a Molecular Switch. Structure.

[22] Mertens J., Casado S., Mata C.P., Hernando-Perez M., de Pablo P.J., Carrascosa J.L., Castón J.R. (2015). A protein with simultaneous capsid scaffolding and dsRNA-binding activities enhances the birnavirus capsid mechanical stability. Sci. Rep..

[23] Dobos P., Hill B.J., Hallett R., Kells D.T., Becht H., Teninges D. (1979). Biophysical and biochemical characterization of five animal viruses with bisegmented double-stranded RNA genomes. J. Virol..

[24] Chevalier C., Galloux M., Pous J., Henry C., Denis J., da Costa B., Navaza J., Lepault J., Delmas B. (2005). Structural Peptides of a Nonenveloped Virus Are Involved in Assembly and Membrane Translocation. J. Virol..

[25] Vakharia V.N., He J., Ahamed B., Snyder D.B. (1994). Molecular basis of antigenic variation in infectious bursal disease virus. Virus Res..

[26] Brandt M., Yao K., Liu M., Heckert R.A., Vakharia V.N. (2001). Molecular determinants of virulence, cell tropism, and pathogenic phenotype of infectious bursal disease virus. J. Virol..

[27] Qi X., Zhang L., Chen Y., Gao L., Wu G., Qin L., Wang Y., Ren X., Gao Y., Gao H. (2013). Mutations of residues 249 and 256 in VP2 are involved in the replication and virulence of infectious Bursal disease virus. PLoS ONE.

[28] Islam M.R., Zierenberg K., Muller H. (2001). The genome segment B encoding the RNA-dependent RNA polymerase protein VP1 of very virulent infectious bursal disease virus (IBDV) is phylogenetically distinct from that of all other IBDV strains. Arch. Virol..

[29] Wei Y., Li J., Zheng J., Xu H., Li L., Yu L. (2006). Genetic reassortment of infectious bursal disease virus in nature. Biochem. Biophys. Res. Commun..

[30] He C.Q., Ma L.Y., Wang D., Li G.R., Ding N.Z. (2009). Homologous recombination is apparent in infectious bursal disease virus. Virology.

[31] Jackwood D.J. (2012). Molecular epidemiologic evidence of homologous recombination in infectious bursal disease viruses. Avian Dis..

[32] He X., Xiong Z., Yang L., Guan D., Yang X., Wei P. (2014). Molecular epidemiology studies on partial sequences of both genome segments reveal that reassortant infectious bursal disease viruses were dominantly prevalent in southern China during 2000–2012. Arch. Virol..

[33] Pitesky M., Cataline K., Crossley B., Poulos M., Ramos G., Willoughby D., Woolcock P., Cutler G., Bland M., Tran J. (2013). Historical, spatial, temporal, and time-space epidemiology of very virulent infectious bursal disease in California: A retrospective study 2008–2011. Avian Dis..

[34] Gallardo R.A., Carrasco-Medanic R., Zhou H., Lyu S., Wang Y., Woolcock P.R., Hoerr F.J. (2014). Effects of challenge with very virulent infectious bursal disease virus reassortants in commercial chickens. Avian Dis..

[35] Kurukulsuriya S., Ahmed K.A., Ojkic D., Gunawardana T., Gupta A., Goonewardene K., Karunaratne R., Popowich S., Willson P., Tikoo S.K. (2016). Circulating strains of variant infectious bursal disease virus may pose a challenge for antibiotic-free chicken farming in Canada. Res. Vet. Sci..

[36] Hiraga M., Nunoya T., Otaki Y., Tajima M., Saito T., Nakamura T. (1994). Pathogenesis of highly virulent infectious bursal disease virus infection in intact and bursectomized chickens. J. Vet. Med. Sci..

[37] McFerran J.B., McNulty M.S., McKillop E.R., Connor T.J., McCracken R.M., Collins D.S., Allan G.M. (1980). Isolation and serological studies with infectious bursal disease viruses from fowl, turkeys and ducks: Demonstration of a second serotype. Avian Pathol..

[38] Kasanga C.J., Yamaguchi T., Wambura P.N., Munang’Andu H.M., Ohya K., Fukushi H. (2008). Detection of infectious bursal disease virus (IBDV) genome in free-living pigeon and guinea fowl in Africa suggests involvement of wild birds in the epidemiology of IBDV. Virus Genes.

[39] Jayasundara J.M., Walkden-Brown S.W., Katz M.E., Islam A.F., Renz K.G., McNally J., Hunt P.W. (2016). Pathogenicity, tissue distribution, shedding and environmental detection of two strains of IBDV following infection of chickens at 0 and 14 days of age. Avian Pathol..

[40] Aricibasi M., Jung A., Heller E.D., Rautenschlein S. (2010). Differences in genetic background influence the induction of innate and acquired immune responses in chickens depending on the virulence of the infecting infectious bursal disease virus (IBDV) strain. Vet. Immunol. Immunopathol..

[41] Tippenhauer M., Heller D.E., Weigend S., Rautenschlein S. (2013). The host genotype influences infectious bursal disease virus pathogenesis in chickens by modulation of T cells responses and cytokine gene expression. Dev. Comp. Immunol..

[42] Bumstead N., Reece R.L., Cook J.K. (1993). Genetic differences in susceptibility of chicken lines to infection with infectious bursal disease virus. Poult. Sci..

[43] Nielsen O.L., Sorensen P., Hedemand J.E., Laursen S.B., Jorgensen P.H. (1998). Inflammatory response of different chicken lines and B haplotypes to infection with infectious bursal disease virus. Avian Pathol..

[44] Sa E.S.M., Rissi D.R., Swayne D.E. (2016). Very Virulent Infectious Bursal Disease Virus Produces More-Severe Disease and Lesions in Specific-Pathogen-Free (SPF) Leghorns Than in SPF Broiler Chickens. Avian Dis..

[45] Inoue M., Yamamoto H., Matuo K., Hihara H. (1992). Susceptibility of chicken monocytic cell lines to infectious bursal disease virus. J. Vet. Med. Sci..

[46] Liang J., Yin Y., Qin T., Yang Q. (2015). Chicken bone marrow-derived dendritic cells maturation in response to infectious bursal disease virus. Vet. Immunol. Immunopathol..

[47] Moyer C.L., Nemerow G.R. (2011). Viral weapons of membrane destruction: Variable modes of membrane penetration by non-enveloped viruses. Curr. Opin. Virol..

[48] Ogawa M., Yamaguchi T., Setiyono A., Ho T., Matsuda H., Furusawa S., Fukushi H., Hirai K. (1998). Some characteristics of a cellular receptor for virulent infectious bursal disease virus by using flow cytometry. Arch. Virol..

[49] Luo J., Zhang H., Teng M., Fan J.M., You L.M., Xiao Z.J., Yi M.L., Zhi Y.B., Li X.W., Zhang G.P. (2010). Surface IgM on DT40 cells may be a component of the putative receptor complex responsible for the binding of infectious bursal disease virus. Avian Pathol..

[50] Lin T.W., Lo C.W., Lai S.Y., Fan R.J., Lo C.J., Chou Y.M., Thiruvengadam R., Wang A.H., Wang M.Y. (2007). Chicken heat shock protein 90 is a component of the putative cellular receptor complex of infectious bursal disease virus. J. Virol..

[51] Delgui L., Ona A., Gutierrez S., Luque D., Navarro A., Caston J.R., Rodriguez J.F. (2009). The capsid protein of infectious bursal disease virus contains a functional alpha 4 beta 1 integrin ligand motif. Virology.

[52] Rose D.M., Han J., Ginsberg M.H. (2002). α4 integrins and the immune response. Immunol. Rev..

[53] Ye C., Han X., Yu Z., Zhang E., Wang L., Liu H. (2016). Infectious Bursal Disease Virus activates c-Src to promote α4β1 integrin-dependent viral entry via modulating downstream Akt-RhoA GTPase-actin rearrangement cascade. J. Virol..

[54] Gimenez M.C., Rodriguez A.J., Colombo M.I., Delgui L.R. (2015). Infectious bursal disease virus uptake involves macropinocytosis and trafficking to early endosomes in a Rab5-dependent manner. Cell. Microbiol..

[55] Galloux M., Libersou S., Morellet N., Bouaziz S., Da C.B., Ouldali M., Lepault J., Delmas B. (2007). Infectious bursal disease virus, a non-enveloped virus, possesses a capsid-associated peptide that deforms and perforates biological membranes. J. Biol. Chem..

[56] Yip C.W., Hon C.C., Zeng F., Leung F.C. (2012). Cell culture-adapted IBDV uses endocytosis for entry in DF-1 chicken embryonic fibroblasts. Virus Res..

[57] Gerke V., Creutz C.E., Moss S.E. (2005). Annexins: Linking Ca2+ signalling to membrane dynamics. Nat. Rev. Mol. Cell. Biol..

[58] Futter C.E., White I.J. (2007). Annexins and endocytosis. Traffic.

[59] Tebar F., Gelabert-Baldrich M., Hoque M., Cairns R., Rentero C., Pol A., Grewal T., Enrich C. (2014). Annexins and endosomal signaling. Meth. Enzymol..

[60] Ren X., Zhang L., Gao Y., Gao H., Wang Y., Liu C., Cui H., Zhang Y., Jiang L., Qi X. (2015). Binding chicken Anx2 is beneficial for infection with infectious bursal disease virus. Virus Res..

[61] Vasconcelos A.C., Lam K.M. (1994). Apoptosis induced by infectious bursal disease virus. J. Gen. Virol..

[62] Tham K.M., Moon C.D. (1996). Apoptosis in cell cultures induced by infectious bursal disease virus following in vitro infection. Avian Dis..

[63] Shahsavandi S., Ebrahimi M.M., Sadeghi K., Mahravani H. (2014). Apoptotic response of chicken embryonic fibroblast cells to infectious bursal disease virus infections reflects viral pathogenicity. In Vitro Cell. Dev. Biol. Anim..

[64] Nguyen M.L., Blaho J.A. (2007). Apoptosis during herpes simplex virus infection. Adv. Virus Res..

[65] Koyama A.H., Adachi A., Irie H. (2003). Physiological significance of apoptosis during animal virus infection. Int. Rev. Immunol..

[66] Lombardo E., Maraver A., Espinosa I., Fernandez-Arias A., Rodriguez J.F. (2000). VP5, the nonstructural polypeptide of infectious bursal disease virus, accumulates within the host plasma membrane and induces cell lysis. Virology.

[67] Fernandez-Arias A., Martinez S., Rodriguez J.F. (1997). The major antigenic protein of infectious bursal disease virus, VP2, is an apoptotic inducer. J. Virol..

[68] Busnadiego I., Maestre A.M., Rodriguez D., Rodriguez J.F. (2012). The infectious bursal disease virus RNA-binding VP3 polypeptide inhibits PKR-mediated apoptosis. PLoS ONE.

[69] Zhang L., Hu T., Liu H., Shuai X. (2011). Inhibitory effect of *Sargassum* polysaccharide on oxidative stress induced by infectious bursa disease virus in chicken bursal lymphocytes. Int. J. Biol. Macromol..

[70] Li G., Scull C., Ozcan L., Tabas I. (2010). NADPH oxidase links endoplasmic reticulum stress, oxidative stress, and PKR activation to induce apoptosis. J. Cell Biol..

[71] Pyo C., Lee S., Choi S. (2008). Oxidative stress induces PKR-dependent apoptosis via IFN-gamma activation signaling in Jurkat T cells. Biochem. Biophys. Res. Commun..

[72] Liu M., Vakharia V.N. (2006). Nonstructural protein of infectious bursal disease virus inhibits apoptosis at the early stage of virus infection. J. Virol..

[73] Qin Y., Xu Z., Wang Y., Li X., He Y., Cao H., Zheng S.J. (2017). VP2 of the infectious bursal disease virus is a protease that induces apoptosis by cleaving the oral cancer overexpressed 1 protein.

[74] Li M., Cui X., Shen Y., Dong H., Liang W., Chen Y., Hu J., Li S., Kong J., Li H. (2015). ORAOV1 overexpression in esophageal squamous cell carcinoma and esophageal dysplasia: A possible biomarker of progression and poor prognosis in esophageal carcinoma. Hum. Pathol..

[75] Jiang L., Zeng X., Wang Z., Ji N., Zhou Y., Liu X., Chen Q. (2010). Oral cancer overexpressed 1 (ORAOV1) regulates cell cycle and apoptosis in cervical cancer HeLa cells. Mol. Cancer.

[76] Togashi Y., Arao T., Kato H., Matsumoto K., Terashima M., Hayashi H., de Velasco M.A., Fujita Y., Kimura H., Yasuda T. (2014). Frequent amplification of ORAOV1 gene in esophageal squamous cell cancer promotes an aggressive phenotype via proline metabolism and ROS production. Oncotarget.

[77] Zhai C., Li Y., Mascarenhas C., Lin Q., Li K., Vyrides I., Grant C.M., Panaretou B. (2014). The function of ORAOV1/LTO1, a gene that is overexpressed frequently in cancer: Essential roles in the function and biogenesis of the ribosome. Oncogene.

[78] Yao K., Vakharia V.N. (2001). Induction of Apoptosis in Vitro by the 17-kDa Nonstructural Protein of Infectious Bursal Disease Virus: Possible Role in Viral Pathogenesis. Virology.

[79] Yao K., Goodwin M.A., Vakharia V.N. (1998). Generation of a mutant infectious bursal disease virus that does not cause bursal lesions. J. Virol..

[80] Li Z., Wang Y., Xue Y., Li X., Cao H., Zheng S.J. (2012). Critical Role for Voltage-Dependent Anion Channel 2 in Infectious Bursal Disease Virus-Induced Apoptosis in Host Cells via Interaction with VP5. J. Virol..

[81] Lin W., Zhang Z., Xu Z., Wang B., Li X., Cao H., Wang Y., Zheng S.J. (2015). The Association of Receptor of Activated Protein Kinase C 1 (RACK1) with Infectious Bursal Disease Virus Viral Protein VP5 and Voltage-dependent Anion Channel 2 (VDAC2) Inhibits Apoptosis and Enhances Viral Replication. J. Biol. Chem..

[82] Wei L., Hou L., Zhu S., Wang J., Zhou J., Liu J. (2011). Infectious bursal disease virus activates the phosphatidylinositol 3-kinase (PI3K)/Akt signaling pathway by interaction of VP5 protein with the p85alpha subunit of PI3K. Virology.

[83] Qin L., Qi X., Gao Y., Gao H., Lu X., Wang Y., Bu Z., Wang X. (2010). VP5-deficient mutant virus induced protection against challenge with very virulent infectious bursal disease virus of chickens. Vaccine.

[84] Palmquist J.M., Khatri M., Cha R.M., Goddeeris B.M., Walcheck B., Sharma J.M. (2006). In vivo activation of chicken macrophages by infectious bursal disease virus. Viral Immunol..

[85] Ragland W.L., Novak R., El-Attrache J., Savic V., Ester K. (2002). Chicken anemia virus and infectious bursal disease virus interfere with transcription of chicken IFN-α and IFN-γ mRNA. J. Interferon Cytokine Res..

[86] Li Z., Wang Y., Li X., Li X., Cao H., Zheng S.J. (2013). Critical roles of glucocorticoid-induced leucine zipper in infectious bursal disease virus (IBDV)-induced suppression of type I Interferon expression and enhancement of IBDV growth in host cells via interaction with VP4. J. Virol..

[87] Di Marco B., Massetti M., Bruscoli S., Macchiarulo A., di Virgilio R., Velardi E., Donato V., Migliorati G., Riccardi C. (2007). Glucocorticoid-induced leucine zipper (GILZ)/NF-κB interaction: Role of GILZ homo-dimerization and C-terminal domain. Nucleic Acids Res..

[88] Wang Y.Y., Liu L.J., Zhong B., Liu T.T., Li Y., Yang Y., Ran Y., Li S., Tien P., Shu H.B. (2010). WDR5 is essential for assembly of the VISA-associated signaling complex and virus-triggered IRF3 and NF-κB activation. Proc. Natl. Acad. Sci. USA.

[89] Barnes P.J., Karin M. (1997). Nuclear factor-κB: A pivotal transcription factor in chronic inflammatory diseases. N. Engl. J. Med..

[90] Akira S., Uematsu S., Takeuchi O. (2006). Pathogen recognition and innate immunity. Cell.

[91] Mittelstadt P.R., Ashwell J.D. (2001). Inhibition of AP-1 by the glucocorticoid-inducible protein GILZ. J. Biol. Chem..

[92] Ye C., Jia L., Sun Y., Hu B., Wang L., Lu X., Zhou J. (2014). Inhibition of antiviral innate immunity by birnavirus VP3 protein via blockage of viral double-stranded RNA binding to the host cytoplasmic RNA detector MDA5. J. Virol..

[93] Wu B., Hur S. (2015). How RIG-I like receptors activate MAVS. Curr. Opin. Virol..

[94] He C., Klionsky D.J. (2009). Regulation mechanisms and signaling pathways of autophagy. Annu. Rev. Genet..

[95] Puleston D.J., Simon A.K. (2014). Autophagy in the immune system. Immunology.

[96] Hu B., Zhang Y., Jia L., Wu H., Fan C., Sun Y., Ye C., Liao M., Zhou J. (2015). Binding of the pathogen receptor HSP90AA1 to avibirnavirus VP2 induces autophagy by inactivating the AKT-MTOR pathway. Autophagy.

[97] Sato S., Fujita N., Tsuruo T. (2000). Modulation of Akt kinase activity by binding to Hsp90. Proc. Natl. Acad. Sci. USA.

[98] Tacken M.G., Thomas A.A., Peeters B.P., Rottier P.J., Boot H.J. (2004). VP1, the RNA-dependent RNA polymerase and genome-linked protein of infectious bursal disease virus, interacts with the carboxy-terminal domain of translational eukaryotic initiation factor 4AII. Arch. Virol..

[99] Stricker R.L., Behrens S.E., Mundt E. (2010). Nuclear factor NF45 interacts with viral proteins of infectious bursal disease virus and inhibits viral replication. J. Virol..

[100] Zhang L., Ren X., Chen Y., Gao Y., Wang N., Lu Z., Gao L., Qin L., Wang Y., Gao H. (2015). Chondroitin sulfate *N*-acetylgalactosaminyltransferase-2 contributes to the replication of infectious bursal disease virus via interaction with the capsid protein VP2. Viruses.

[101] Wang N., Zhang L., Chen Y., Lu Z., Gao L., Wang Y., Gao Y., Gao H., Cui H., Li K. (2015). Cyclophilin A Interacts with Viral VP4 and Inhibits the Replication of Infectious Bursal Disease Virus. BioMed Res. Int..

